# Shared decision making PLUS – a cluster-randomized trial with inpatients suffering from schizophrenia (SDM-PLUS)

**DOI:** 10.1186/s12888-017-1240-3

**Published:** 2017-02-23

**Authors:** Johannes Hamann, Fabian Holzhüter, Lynne Stecher, Stephan Heres

**Affiliations:** 1 Klinik und Poliklinik für Psychiatrie und Psychotherapie, Technische Universität München, Ismaninger Straße 22, 81675 Munich, Germany; 2 Institut für Medizinische Statistik und Epidemiologie, Klinikum rechts der Isar der TU München, Ismaninger Str. 22, 81675 Munich, Germany

**Keywords:** Shared decision making, Schizophrenia, Adherence

## Abstract

**Background:**

Shared decision making (SDM) is a model of how doctors and patients interact with each other. It aims at changing the traditional power asymmetry between doctors and patients by strengthening the exchange of information and the decisional position of the patient. Although SDM is generally welcomed by mental health patients as well as by mental health professionals its implementation in routine care, especially in the more acute settings, is still lacking. SDM-PLUS has been developed as an approach that addresses both patients and mental health professionals and aims at implementing SDM even for the very acutely ill patients.

**Methods:**

The SDM-PLUS study will be performed as a matched-pair cluster-randomized trial in acute psychiatric wards. On wards allocated to the intervention group personnel will receive communication training (addressing how to implement SDM for various scenarios) and patients will receive a group intervention addressing patient skills for SDM. Wards allocated to the control condition will continue treatment as usual. A total sample size of 276 patients suffering from schizophrenia or schizoaffective disorder on 12 wards is planned.

The main outcome parameter will be patients’ perceived involvement in decision making during the inpatient stay measured with the SDM-Q-9 questionnaire. Secondary objectives include the therapeutic relationship and long term outcomes such as medication adherence and rehospitalization rates. In addition, process measures and qualitative data will be obtained to allow for the analysis of potential barriers and facilitators of SDM-PLUS.

The primary analysis will be a comparison of SDM-Q-9 sum scores 3 weeks after study inclusion (or discharge, if earlier) between the intervention and control groups. To assess the effect of the intervention on this continuous primary outcome, a random effects linear regression model will be fitted with ward (cluster) as a random effect term and intervention group as a fixed effect.

**Discussion:**

This will be the first trial examining the SDM-PLUS approach for patients with schizophrenia or schizoaffective disorder in very acute mental health inpatient settings. Within the trial a complex intervention will be implemented that addresses both patients and health care staff to yield maximum effects.

**Trial registration:**

German Clinical Trials Register DRKS00010880. Registered 09 August 2016.

## Background

Shared decision making (SDM) is a model of how doctors and patients interact with each other. It aims at changing the traditional power asymmetry between doctors and patients by strengthening the exchange of information and the decisional position of the patient [1]. While some see the need for SDM from an ethical point of view [2], others argue that SDM may contribute to better patient satisfaction and health outcomes [3]. Some of the basic principles of SDM aim at making the decision process more explicit by inviting patients to participate and by clarifying information and participation preferences. Amongst others, the strategies involve stating that there is more than one option to choose from (‘equipoise’), communicating the pros and cons of different options and helping patients build their own preferences (e.g. by administering decision aids) [4, 5].

Although SDM originates from somatic medicine, its ideas and ideals may be especially important for the field of mental health where the possibility of involuntary treatment often creates extreme forms of ‘power asymmetry’, where the importance of long-term adherence requires special attention for patient satisfaction with the treatment and where the patients’ potentially handicapped decision-making capacity may lead doctors to avoid practicing SDM [2, 6].

In fact, there is strong evidence that SDM is actually not really implemented in various mental health settings [7, 8]. While low implementation of SDM is not unique for mental health it may be less often implemented compared to other medical fields [9].

Besides the well-known general barriers to SDM [10] (e.g. time constraints), there may be mental health specific factors (or factors that may be more pronounced in metal health settings) that hinder the implementation of SDM.

On the patients’ side, these barriers include, among others, a lack of interest in decision making (e.g. due to depressive or negative symptoms) [6, 11], a feeling of powerlessness in relationship with their providers [11], or passivity in the medical encounter [12]. On the psychiatrists’ side, reservations to implement SDM are often founded in doubts regarding patients’ insight and decisional capacity [11, 13, 14]. Here, psychiatrists might be afraid to make patient outcomes worse by sharing decisions and thereby reaching unreasonable decisions.

The authors have therefore developed a framework for the implementation of SDM (shared decision making PLUS, SDM-PLUS [15]) that specifically addresses these barriers by empowering both patients and providers.

## Methods

### Rationale

As stated above SDM is not routinely implemented in clinical practice [9, 13] and research on SDM-interventions in mental health setting is limited. Most single interventions (e.g. SDM training for GPs [16], computerized SDM-tool [17], web-based tool to support shared decision-making [18]) have yielded only small effects at best.

Therefore, we argue that it will need a complex intervention (“defined as intervention(s) with several interacting components” [19]) such as SDM-PLUS addressing both, patients and health care providers to change patterns in medical decision making towards a more participatory atmosphere, especially on acute psychiatric wards. While the complexity of the intervention is explicitly desired with respect to a strengthening of effects, we have to acknowledge at the same time the drawbacks of complex interventions (e.g. the impossibility to trace back effects to single compounds of the intervention) [20].

To account for these limitations, we will apply a mixed-methods approach to study process measures and potential modes of action of the intervention (see methods for details).

### Aim and hypotheses

**Fig. 1:**
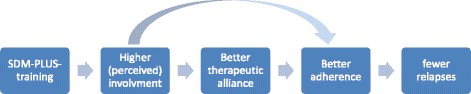
Potential outcomes of SDM-PLUS

The aim of the present study is to evaluate the effects of SDM-PLUS on decision making patterns on acute psychiatric wards between psychiatrists and patients suffering from schizophrenia. We hypothesize that the intervention will lead to professionals and patients using the skills learned in the SDM-PLUS training for professionals or the SDM-training for patients, respectively, leading to a higher perceived involvement of patients. We argue that a better (perceived) involvement of patients in medical decisions would be a benefit per se [2]. However, a higher perceived involvement from the patients’ side may also have an effect on the therapeutic alliance finally resulting in better adherence and fewer relapses caused by non-adherence [21, 22] (Fig. 1).

The primary objective is to assess if there is a group difference (intervention vs. control) in patients’ perceived involvement in decision making 3 weeks after study enrollment (or discharge from the ward, whatever happens first) using the SDM-Q-9 questionnaire [23].

### Trial design

**Fig. 2:**
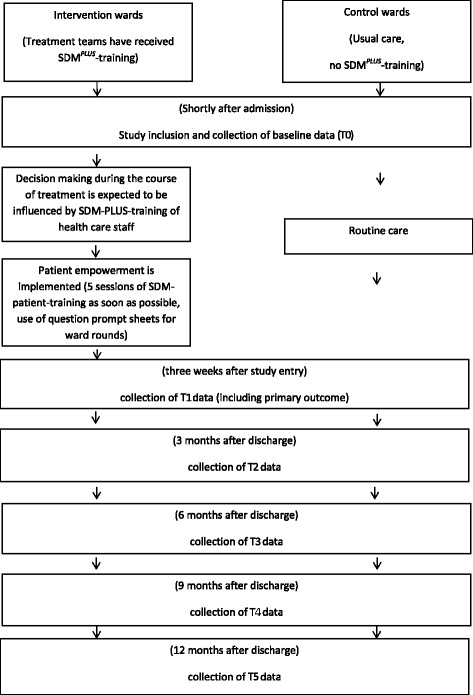
Timeline chart

The study is designed as a multi-center, matched-pair cluster-randomized controlled trial of SDM-PLUS in acute psychiatric wards addressing inpatients suffering from schizophrenia or schizoaffective disease. SDM-PLUS will be implemented in the intervention wards while on the control wards treatment will be continued as usual (Fig. 2).

### Participants

The study will be implemented in 12 acute psychiatric wards (=clusters) of 5 psychiatric state hospitals. Eligibility criteria for the wards to be included are: acute psychiatric ward in one of the participating hospitals, patients with schizophrenia or schizoaffective disease are commonly treated on these wards. The respective wards have 300–600 admissions per year of which approximately 20–50% of patients meet inclusion criteria.

All patients fulfilling the inclusion criteria will then be consecutively recruited for the trial at the time of their admission to the ward.

Inclusion criteria:

- age 18–65 years
- male and female patients
- inpatients of participating hospitals
- diagnosis of schizophrenia or schizoaffective disease (ICD 10: F20/F25)
- capable of participating in 60 min. group intervention
- being able to provide written informed consent

Exclusion criteria:

- mental retardation
- insufficient proficiency in German language to discuss treatment decisions

### Intervention and control condition

Treatment teams (consultants, residents, nurses, psychologists and social workers) of intervention wards will be trained in the SDM-PLUS-approach through participation in two half-day workshops (within 2 weeks) and will then be continuously supervised and supported by the study center over the entire course of the trial (Fig. 2). The supervision will take the form of weekly meetings with the physicians in charge. Within these meetings, a checklist will be completed for every patient currently participating in the study to review what has been done to facilitate SDM for this patient (see also the section “treatment integrity”).

The SDM-PLUS-approach [15] has two basic aims: to empower health care staff (communication techniques) and to teach health care staff how to empower patients (implementation of empowering measures for patients). Empowerment training for health care staff includes decision analysis and communication strategies about how to involve patients in medical decisions. Decision analysis means that health care professionals must analyze how to best reach shared decision making in every individual patient. SDM-PLUS proposes that there are situations in which SDM can be reached via the “classical way” (i.e. by discussion pros and cons of several more or less equivalent options) and that there are situations for which techniques other than “classical” shared decision making must be used to reach joint decisions, especially when there is resistance from the patient’s side. Here, techniques from the Harvard negotiation model and from motivational interviewing are taught. Participants of SDM-PLUS workshops will be presented with “ideal” courses of action for all three approaches (classical SDM, preparing for difficult negotiations according to the Harvard model and reflective listening as an important aspect of motivational interviewing) and then will exercise these techniques in supervised role plays.

There is special emphasis on how to successfully address “difficult decisions” and avoid underutilization of effective treatment interventions because of non-addressing these techniques in discussions with the patient.

The second aim of SDM-PLUS is to empower patients to be more active partners in medical decision making. Measures will consist of patient group training in SDM and the use of question prompt sheets for ward rounds and individual consultations. The SDM training will be implemented as a five-session group training [24] (2 sessions per week) that will be led by a member of the study center and a member of the respective treatment team to ensure proper implementation. Within the training patients will be made familiar with the concept and prospects of SDM and the basic communication skills necessary to facilitate SDM from the patients’ side. Thus patients will work out the importance of preparing for ward rounds, prompting questions and expressing opinions and exercise these skills within role plays and home works. The SDM training will be offered as an open group to ensure that patients newly recruited to the study can immediately participate. The question prompt sheets will be promoted within the SDM training and implemented on the wards by nursing staff.

Staff (and patients) of the control wards will act under “treatment as usual” (TAU) conditions but will be offered SDM-PLUS-training after the end of the study. In order to minimize contamination bias (i.e. staff or patients from control wards getting to know about SDM-PLUS), wards were selected to ensure that there is no overlap in personnel and no regular patient transfer between wards.

### Outcomes

At all time-points identical data will be collected in the intervention and control groups (Fig. 2). In our study we will follow an intention to treat (ITT) approach. Therefore we will try to follow up all participants in the 12 months periods following hospital discharge irrespectively of whether they are still in treatment or on medication. We seek for patients’ permission to contact them directly as well as their actual physicians (if applicable) at 3/6/9/12 months. By applying this 2-way-approch we will also be able to approach patients who no longer receive any kind of psychiatric treatment.

#### Baseline parameters

For all patients enrolled socio-demographics, diagnosis, illness severity (CGI and GAF scores) and data on illness history (previous hospitalizations, duration of illness etc.) will be recorded at baseline (at study entry).

Since decision making and patients’ perception of decision making may be influenced by patients’ participation preferences, the Autonomy Preference Index (API [25]) will be obtained. The API is a four item questionnaire assessing patients’ general desire to participate in decision making. API scores have been shown to be associated with treatment satisfaction and health care outcomes [26]. Further, a recently developed questionnaire addressing patients’ competence for shared decision making (PatPart-19) will also be used. This 19 item questionnaire covers the subscales open communication, critical communication and adherence in therapy and was validated in two large samples of psychiatric inpatients (Kohl et al., in preparation). In addition, active patient decision making has been shown to be related to patients’ having less unmet needs [27]. Therefore, the Camberwell Assessment of Need self-report questionnaire (CANSAS-P), a 22-item questionnaire addressing patients’ needs for support in various domains will be applied. As SDM-PLUS focusses on critical decision processes in which disagreement between patients and physicians may occur we aim to identify patients with potentially reduced insight, allowing for subgroup analyses in patients with or without insight. We will administer the Birchwood Insight Scale [28], an eight-item questionnaire addressing insight, necessity of medication or treatment. Likewise patients’ perception of the current admission (i.e. the extent to which patients perceived the admission as involuntary) will be recorded using the MacArthur Admission Experience Survey as used by O’Donoghue et al. 2013 [29].

#### Primary outcome

The primary outcome parameter is the patients’ perceived involvement in decision making using the SDM-Q-9 questionnaire at 3 weeks after enrollment in the study or discharge (whatever occurs first). Referring to Rodenburg-Vandenbussche et al. [30] we see a 15 point difference as clinically meaningful (in the mentioned study this 15- point difference differentiated between shared decision making and physician dominated decision making as perceived by patients) and we expect a 15 point mean difference between the intervention and control group (Table 1). The SDM-Q-9 refers to a distinct medical decision which will be the patient’s medication regime, which in many cases is the result of several smaller decisions during inpatient stay and thereby reflecting the decision atmosphere between patients and their treating physicians. Therefore the SDM-Q-9 will serve as a proxy for inpatient decision making.

**Table 1:**
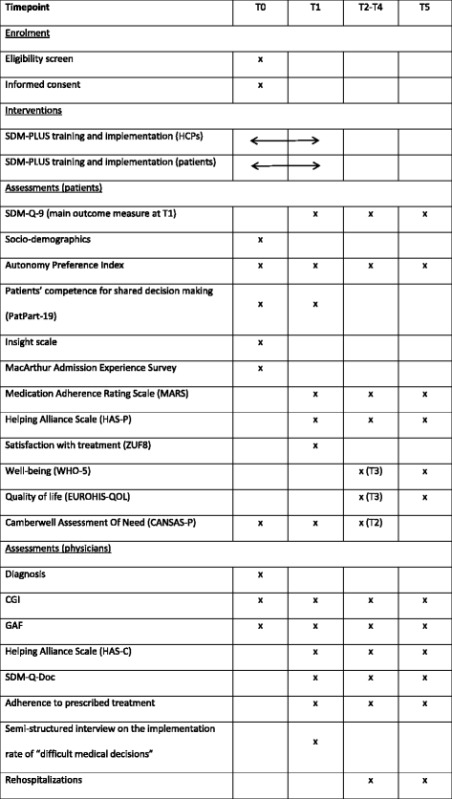
Planned data collection

#### Secondary outcomes

As outlined above, a better involvement of patients in medical decisions and health care professionals better addressing “difficult decisions” may also result in a better therapeutic relationship, higher treatment satisfaction, fewer unmet needs, more thoroughly implemented therapies, improved adherence, and probable reduced relapse rates (see Fig. 1). These outcomes will be obtained at T1 (three weeks after study enrolment) and during a one year follow up with quarterly assessments (T2-T5).

Patients’ involvement in medical decisions from the patients’ point of view will be obtained as the primary outcome measure at T1 and as a secondary outcome during follow up (T2-T5). In addition, physicians will provide their view on patients’ involvement using the physician version of the SDM-Q-9 at T2-T5 (SDM-Q-Doc [31]). As most patients will have changes in their treating psychiatrist when switching from inpatient to outpatient treatment, the SDM-Q-9 refers to the inpatient psychiatrist at T1 and to the outpatient psychiatrists at T2-T5.

Whether or not the intervention also has an influence on the therapeutic relationship will be determined using the Helping Alliance Scale at T2-T5, which has a patient (HAS-P) and a clinician (HAS-C) version [32]. The HAS comprises five items rated on a visual analogue scale ranging from 0 (‘not at all’) to 10 (‘extremely well’). While HAS-P includes items on ‘right treatment’, ‘understood by therapist’, ‘criticized by therapist’, ‘committed therapist’ and ‘trust therapist, HAS-C items cover ‘getting along with patient’, ‘understand patient’, ‘look forward to meeting patient’, ‘feel actively involved’, and ‘feel I can help patient’.

Treatment satisfaction will be measured using the Questionnaire on Patients’ Treatment Satisfaction (ZUF8) at T1, an eight-item questionnaire addressing general satisfaction with hospital care [33]. The prevalence of unmet needs on the patients’ side after intervention will be assessed using the Camberwell Assessment of Need self-report questionnaire (CANSAS-P) also at T1 and T2 [34]. For the assessment of adherence, patients will fill out the Medication Adherence Rating Scale at T2–T5 (MARS [35]). This measure also includes the 10 items of the Drug Attitude Inventory allowing for an analysis of patients’ drug attitudes.

In addition, aspects of patients’ well-being and quality of life will be addressed using the WHO-5 well-being index and the EUROHIS-QOL index at T3 and T5. The WHO-5 index is a five item self-report measure of general well-being and the EUROHIS-QOL is an 8-item self-assessment instrument of generic quality of life. Both instruments were validated in German versions and yielded good to excellent psychometric properties [36].

Another aspect of interest is whether or not SDM-PLUS supports clinicians in addressing “difficult medical decisions”. This assumption is based on evidence that the discussion of certain decisions (e.g. long acting antipsychotic injectable) is often avoided by psychiatrists resulting in low implementation rates for these treatments [37]. To document the discussion and implementation of “difficult medical decisions” we will perform a semi-structured interview with the psychiatrists in charge at T1. This interview will – for every patient participating in the study - cover four domains: “difficult compounds”, “difficult other therapies such as ECT”, “difficult psychosocial interventions such as legal guardianship”, and “difficult other decisions as specified by the psychiatrist”. For each domain it will be documented whether or not there was any need for discussing decisions, whether there was a discussion and whether any decisions were implemented. Data gathered in this interview will result in two “sum scores” of difficult decisions (number of decisions addressed, number of decisions implemented).

In addition, we will obtain at discharge, the length of the inpatient stay, the future psychiatrist after discharge and a brief description of discharge medication (antipsychotics prescribed: yes/no, LAI prescribed: yes/no, monotherapy with antipsychotics vs polypharmacy with antipsychotics: yes/no). Outpatient psychiatrists will be asked to document any rehospitalization of their patients, their estimate of the patient’s adherence (one item visual analogue scale), CGI and GAF scores at T2–T5.

#### Qualitative data

The rationale behind also obtaining qualitative data is twofold. First, we would like to get a better insight into the process and working mode of SDM-PLUS and, second, we aim to study the potential barriers and facilitators of SDM-PLUS.

Therefore, we will use the critical incident method to identify clinical situations of interest (e.g. dissatisfied/very satisfied patients, decisions with or without patient participation, patients for whom health staff judges SDM-PLUS as inappropriate or impossible etc.) and then perform qualitative interviews with 24 key informants involved in the trial (physicians, nurses, patients). In addition, we will perform focus groups with health care staff after the end of the study to collect their experiences with the intervention. The qualitative part of the study will take place only on intervention wards and will be performed by two trained researchers who are working for the project and are in permanent contact with patients/staff of the participating hospitals.

The qualitative data will be audio-recorded, transcribed and then analyzed using content analysis [38].

#### Treatment integrity

To assess treatment adherence we will document: a) how many of the team members actually receive the SDM-PLUS training, b) whether there was a change in attitudes towards SDM (pre-/post-questionnaire), and c) to what extent single elements of the intervention are implemented for patients (i.e. use of a “fidelity scale” during weekly supervision with all physicians in charge). On the patients’ side, the number of patient intervention sessions per patient and the percentage of patients receiving question prompt sheets will be documented.

In addition, we will use a qualitative approach in the intervention group to assess various aspects of the process of SDM-PLUS as well as barriers and facilitators of SDM-PLUS (see “Qualitative data”).

### Sample size

The primary analysis will be a comparison of SDM-Q-9 sum scores at T1 between the intervention and control groups. Referring to Rodenburg-Vandenbussche et al. 2015 [30], a 15 point difference (SD 30) is considered clinically meaningful. For the study to have 80% power to detect this mean difference, 23 patients will be recruited in 12 wards, giving a total of 276 patients. This calculation assumes a two-sided significance level of 5%, a within-cluster standard deviation of 30, intra-cluster coefficient of 0.04 and 20% dropout.

### Randomization

We will determine pairs of comparable wards (number of patients, distribution of diagnoses, staff etc.) and then randomize one ward of each pair to the intervention and one ward to the control condition (i.e. cluster-randomization). While the principal investigators will determine the paired wards, the randomization will be done by the statistical department of the medical faculty at our university, Institut für Medizinische Statistik und Epidemiologie (IMSE).

A cluster-randomized design in which the unit of randomization is the psychiatric ward is seen as necessary to prevent contamination of intervention and control conditions [19].

### Blinding

Due to the nature of the intervention (staff training, patient training) there will be no blinding. Likewise a blinding of raters is not applicable because most ratings, including the main outcome measure, are “subjective” self-ratings. However, the analysis will be performed by a statistician who will be blinded as to patient allocation.

To avoid selection bias all patients fulfilling inclusion criteria will be recruited consecutively in the intervention and control group. This process will be monitored by the study center and will be reported in form of a CONSORT diagram. The number of patients who do not consent will be carefully documented together with their underlying reason for refusal.

### Statistical analyses

To assess the effect of the intervention on the continuous primary outcome, a random effects linear regression model will be fitted with ward (cluster) as a random effect term and intervention group as a fixed effect. The point estimate for the intervention effect will be reported together with the corresponding 95% confidence interval. A *p*-value <0.05 will be considered as providing statistical significant evidence of a group difference. In addition, models adjusting for baseline covariates such as severity of illness, admission status and variables with a large baseline imbalance will be fitted in secondary analyses. An intention to treat approach will be taken to the analysis, i.e. patients in intervention clusters will be analyzed in this group (if outcome data are available) even if they don’t receive all of the planned intervention.

Exploratory analyses will be performed to assess the effect of the intervention on the secondary outcome measures. Furthermore, it will be assessed if SDM is a mediator for the secondary outcomes following the criteria of Baron and Kenny. Random effect linear models will be fitted to the continuous secondary outcome measures, analogous to the primary analysis. For binary secondary outcome measures, logistic regression models using GEEs will be fitted.

### Ethics, informed consent procedure and trial registration

The trial has been approved by the local review board (Ethikkommission der Technischen Universität München). All patients will be informed about the general purpose of the trial (i.e. that decision making patterns will be compared between different wards) but not about randomization and about the intervention condition [39] and then asked for their informed consent. Following the trial all patients will be debriefed about the study and the condition they received. The trial has been registered at Deutsches Register Klinischer Studien (DRKS00010880).

## Discussion

To date, several studies have evaluated interventions to promote SDM in mental health settings. However, only few have addressed the very acutely ill patients and most have had negative results or shown only modest effects.

This will be the first trial examining the SDM-PLUS approach for patients with schizophrenia or schizoaffective disorder in very acute mental health inpatient settings. Within the trial a complex intervention will be implemented that addresses both patients and health care staff to yield maximum effects. The primary outcome is patients’ (perceived) involvement in decision making. Since we expect involvement in decision making to improve other outcomes such as therapeutic alliance, adherence and relapse rates, these will be addressed as secondary outcomes.

It would also have been desirable to have observational measures of the doctor-patient-communication, as SDM-PLUS aims at altering communication. However, since we will study inpatients no clearly definable and between intervention and control comparable consultations exist.

Potential methodical limitations may be the extent to which the proposed strategies are actually implemented by health care staff and a recruitment bias. We will aim to minimize these limitations via a thorough assessment of process measures within a mixed-methods approach and through recruiting consecutively on all intervention and control wards. In addition, to avoid for contamination we do not control for time and attention paid to patients in the control group and do not offer an active control condition such as a unspecific group training. This bears the risk that more attention alone may drive any changes in perceived SDM. Finally, the main outcome measure (SDM-Q9) asks for the decision process that led to the current antipsychotic medication. We therefore focus on the patients’ perception of SDM with the prescriber (i.e. psychiatrist) and put less emphasis on patients’ experiences with other staff members.

The major aim is to show that SDM-PLUS, if thoroughly implemented, leads to a higher perceived involvement of patients in medical decision making. While one might argue that involvement of patients alone might not constitute a “clinical” or even financial benefit, we claim that the proof of a better involvement by the introduction of a new communication strategy does have at least two important implications: It fulfils the wish of many users of mental health services to be addressed as competent individuals (as it is ethically necessary anyhow) and it would show that being highly symptomatic does not prevent patients from sharing decisions. Finally, we hope that – via our secondary outcomes – an additional value of ethically correct decision making with respect to a reduction of long term complications (i.e. non-adherence, rehospitalizations) can also be shown.
